# Design of a variant surface antigen-supplemented microarray chip for whole transcriptome analysis of multiple *Plasmodium falciparum *cytoadherent strains, and identification of strain-transcendent *rif *and *stevor *genes

**DOI:** 10.1186/1475-2875-10-180

**Published:** 2011-06-30

**Authors:** Antoine Claessens, Ashfaq Ghumra, Archna P Gupta, Sachel Mok, Zbynek Bozdech, J Alexandra Rowe

**Affiliations:** 1 Centre for Immunity, Infection and Evolution, Institute of Immunology and Infection Research, School of Biological Sciences, University of Edinburgh, West Mains Rd, Edinburgh, EH9 3JT, UK; 2 School of Biological Sciences, Nanyang Technological University, 60 Nanyang Drive, 637551, Singapore

## Abstract

**Background:**

The cytoadherence of *Plasmodium falciparum *is thought to be mediated by variant surface antigens (VSA), encoded by *var*, *rif*, *stevor *and *pfmc-2tm *genes. The last three families have rarely been studied in the context of cytoadherence. As most VSA genes are unique, the variability among sequences has impeded the functional study of VSA across different *P. falciparum *strains. However, many *P. falciparum *genomes have recently been sequenced, allowing the development of specific microarray probes for each VSA gene.

**Methods:**

All VSA sequences from the HB3, Dd2 and IT/FCR3 genomes were extracted using HMMer software. Oligonucleotide probes were designed with OligoRankPick and added to the 3D7-based microarray chip. As a proof of concept, IT/R29 parasites were selected for and against rosette formation and the transcriptomes of isogenic rosetting and non-rosetting parasites were compared by microarray.

**Results:**

From each parasite strain 50-56 *var *genes, 125-132 *rif *genes, 26-33 *stevor *genes and 3-8 *pfmc-2tm *genes were identified. Bioinformatic analysis of the new VSA sequences showed that 13 *rif genes *and five *stevor *genes were well-conserved across at least three strains (83-100% amino acid identity). The ability of the VSA-supplemented microarray chip to detect cytoadherence-related genes was assessed using *P. falciparum *clone IT/R29, in which rosetting is known to be mediated by PfEMP1 encoded by *ITvar9*. Whole transcriptome analysis showed that the most highly up-regulated gene in rosetting parasites was *ITvar9 *(19 to 429-fold up-regulated over six time points). Only one *rif *gene (*IT4rifA_042*) was up-regulated by more than four fold (five fold at 12 hours post-invasion), and no *stevor *or *pfmc-2tm *genes were up-regulated by more than two fold. 377 non-VSA genes were differentially expressed by three fold or more in rosetting parasites, although none was as markedly or consistently up-regulated as *ITvar9*.

**Conclusions:**

Probes for the VSA of newly sequenced *P. falciparum *strains can be added to the 3D7-based microarray chip, allowing the analysis of the entire transcriptome of multiple strains. For the rosetting clone IT/R29, the striking transcriptional upregulation of *ITvar9 *was confirmed, and the data did not support the involvement of other VSA families in rosette formation.

## Background

*Plasmodium falciparum *is the apicomplexan organism causing most malaria deaths. Clinical symptoms occur when the parasite enters the intraerythrocytic cycle. One feature characterising a red blood cell infected with *P. falciparum *at pigmented trophozoite stage is its ability to cytoadhere to human cells. The three main forms of cytoadherence are binding to microvascular endothelial cells, binding to uninfected red blood cells (rosetting) and binding to platelets ("platelet-mediated clumping"), (reviewed in [1]). Sequestration of infected red blood cells (iRBC) in the microvasculature, whether by direct attachment to microvessel walls or indirectly via platelets or red blood cells, is probably a way for parasites to avoid clearance by the spleen [2]. Sequestration in combination with high parasite burdens can, however, cause microvascular obstruction leading to acidosis, hypoxia and release of harmful inflammatory cytokines [3]. Rosetting in particular has been consistently associated with severe malaria cases in sub-Saharan Africa [4-6]. The importance of rosetting in virulent infections is underlined by the observation that human red blood cell polymorphisms that reduce the ability of *P. falciparum *to form rosettes offer substantial protection against life-threatening malaria [7,8].

The parasite molecules thought to mediate cytoadherence are the variant surface antigens (VSA). VSA genes, generally located in the subtelomeric regions, encode proteins exported to the surface of the iRBC. They include the *var*, *rif*, *stevor *and *pfmc-2tm *families, a total of 200 to 300 genes per genome. The *var *gene family, encoding *P. falciparum *Erythrocyte Membrane Protein 1 (PfEMP1), is the most-well studied VSA family (reviewed in [9]). The ~60 *var *genes per isolate can be subdivided into three main groups (A, B or C) based on the upstream regions [10]. The classification has functional and clinical relevance [11-15]. The *var *gene family undergoes mutually exclusive expression, so that a single PfEMP1 variant is found at the surface of the iRBC, although exceptions can occur [16]. Each PfEMP1 is composed of DBL (Duffy Binding-Like) and CIDR (Cysteine rich Inter-Domain Region) domains. Some of these domains have been shown to bind to human cell surface receptors, such as ICAM-1 (by DBLβ), CD36 (by CIDRα, found in group B and C PfEMP1) and red cells via CR1 (DBLα of group A PfEMP1) (reviewed in [1]).

By definition, VSA sequences show low similarity between paralogues, while orthologues cannot be found across strains (one notable exception is *var2csa*, a relatively well conserved *var *gene member) [17]. This implies that VSA sequences are unique to a particular *P. falciparum *strain/isolate. A function assigned to a particular VSA is thus difficult to extrapolate to other *P. falciparum *strains.

So far, virtually all studies investigating the parasite ligands for cytoadherence have focused on *var *genes only. Whether other VSA or non-VSA genes could be involved in adhesion is therefore untested. Moreover, the reference strain 3D7 is often used despite its poor ability to cytoadhere compared to other laboratory strains and field isolates. Recently, many *P. falciparum *genomes, including Dd2, HB3 and IT/FCR3 have been sequenced [18,19]. These three strains are selectable for various cytoadherence phenotypes [20,21] (and JA Rowe, unpublished data). Therefore, to study cytoadherence-related genes in these strains by microarray, it is possible to extract the VSA sequences from a sequenced genome and design oligonucleotide probes specific to each VSA gene. These oligos are added to the 3D7-based microarray chip, allowing the analysis of the full transcriptome of a new strain. As a proof of concept, the IT/R29 strain was selected for and against rosetting and the transcriptome of both populations was analysed with a "VSA-supplemented" microarray chip containing oligos specific for IT VSA. IT/R29 was chosen because it is a well-characterized strain whose rosetting ligand is the group A PfEMP1 variant encoded by the *ITvar9 *gene (also known as *R29var1*) [22,23]. Therefore, the aim of this work was to determine whether the VSA-supplemented microarray chip successfully identified *ITvar9 *as the major up-regulated gene in rosetting parasites, and to use whole transcriptome analysis to identify other candidate genes that could be accessory molecules in rosette formation.

## Methods

**Figure 1:**
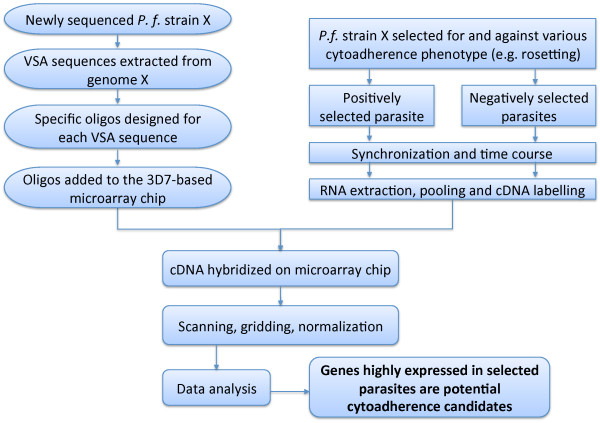
**Summary of the microarray chip design and experimental protocol**. A bioinformatic approach was used to extract VSA sequences and design oligonucleotide probes for a particular strain, while the parasites were selected for and against a cytoadherence phenotype (rosetting). cDNA from positively and negatively selected parasites was hybridized on the "VSA-supplemented" 3D7-based microarray chip in order to identify cytoadherence gene candidates that were up-regulated in rosetting parasites compared to isogenic non-rosetting parasites.

The overall approach used to design a VSA-supplemented microarray chip and investigate the transcriptional profiles of parasites selected for and against a particular cytoadherence phenotype is shown in Figure 1.

### Genome sequences

The HB3 and Dd2 genomes (1189 and 2837 supercontigs, respectively) were downloaded from the Broad institute [18]. The IT/FCR3 genome (2185 supercontigs) was downloaded from the Sanger institute [19]. All other sequences were obtained from PlasmoDB [24].

### Extracting sequences from a genome

**Figure 2:**
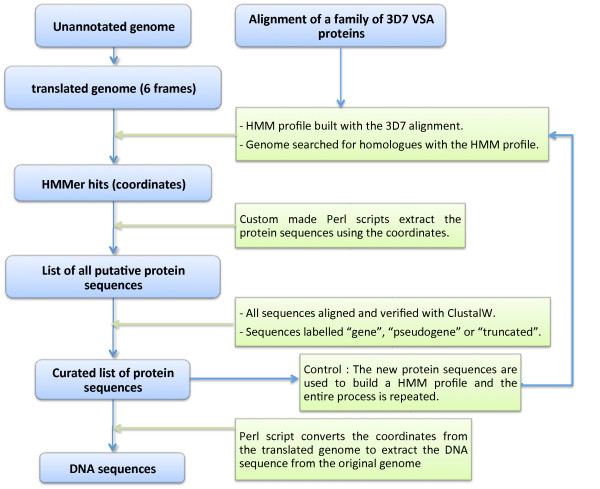
**Flowchart of the process used for extracting VSA sequences from a genome**.

The entire method used for extracting VSA sequences is outlined in Figure 2. The HMMer software, an implementation of profile Hidden Markov Models (HMM), was used with default parameters [25]. In this case, HMMer is used to search for VSA homologues in the HB3/Dd2/IT genomes using an "HMMer profile" based on an alignment of 3D7 sequences. In other words, the programme uses conserved motifs within a given protein family and finds similar motifs in a new genome.

To establish the HMMer profile, each translated exon of each VSA family (*var*, *rif*, *stevor*, *pfmc-2tm*) of the well-annotated 3D7 genome was aligned separately with ClustalW. Each resulting file was used to build a profile HMM specific to that exon. These profiles were used to search for homologues in the six frames of the translated HB3, Dd2 and IT genomes, as well as the *Neurospora crassa *(negative control, genome of similar size) and 3D7 genomes (positive control). The HMMer output file only shows the coordinates of the hits, thus Perl scripts were used to retrieve the actual amino acid sequences from the coordinates. Sequences with stop codon(s) and/or frameshift were annotated as "pseudogene" while shorter sequences with missing amino acids were annotated as "truncated". As a final control, each group of newly derived VSA sequences was used to build a HMM profile and searched against their original genome. If a comprehensive set of VSA had been extracted in the initial procedure, then no new sequences would be expected in the repeated search.

### Designing specific oligos for a microarray chip

70-mer long oligonucleotide probes ("oligos") specific to the extracted VSA sequences were designed using OligoRankPick [26]. Oligos were added onto the 3D7-based microarray chip [27].

### Parasite culture and rosette selection

*Plasmodium falciparum *strain R29 is a clone derived from the IT/FCR3 strain [21] and has been used to study the molecular basis of rosetting [22,28]. R29 was cultured at 2% haematocrit with group A erythrocytes (Scottish National Blood Transfusion Service, Edinburgh, UK) in supplemented RPMI as described elsewhere [29]. Parasitaemia was maintained at 5-10% and cultures were synchronized by sorbitol lysis [30]. Parasites were incubated at 37°C in the presence of 3% CO_2_, 1% O_2 _and 96% N_2 _and routinely screened to avoid mycloplasma contamination [31]. Parasites were replenished with media everyday and fresh erythrocytes every other day. The R29 strain was separated into rosette-positive (R+) and rosette-negative populations (R-) by centrifugation through 60% Percoll or gelatin flotation 2-3 times a week [32]. The rosette frequency of both rosette positive and negative populations was measured by counting the percentage of infected erythrocytes that form rosettes out of 300 infected erythrocytes. Wet preparations of culture suspension stained with 25 mg/ml of ethidium bromide were viewed using a combination of fluorescence and white light. A rosette was defined as an infected erythrocyte that bound two or more uninfected erythrocytes. R29R+ was at a rosette frequency of 73.2% and R29R- was at a rosette frequency of 1.3% at the pigmented trophozoite stage prior to RNA sample collection.

### Synchronisation and time-course experiment

In the three weeks prior to RNA collection, sorbitol lysis was carried out twice at 12 hour intervals in every asexual cycle. For the time course, schizont stage cultures were examined hourly until the first ring was seen, then sorbitol lysis was carried out 12 hours later, giving ring stage parasites within an 12-hour time window. Samples were collected for RNA immediately after sorbitol lysis (time point 1), and then 8-hourly, resulting in 6 time points covering the entire intra-erythrocytic life cycle. For RNA, room temperature TRIzol reagent (Invitrogen 15596-026) was added (ten times the packed cell volume of the cell pellet), and after thorough mixing, stored at -80°C.

### RNA extraction and cDNA synthesis

RNA was extracted as described [33] with minor modifications as follows. The tube with TRIzol solution was thawed on ice. Two volumes of chloroform (Sigma) were added per volume of packed cells. After mixing, the tube was incubated on ice for 5 min then centrifuged at 3600 g for 40 min at 4°C without brakes. The supernatant (aqueous layer) was carefully transferred into a fresh tube without disturbing the interface. The same volume of ice-cold isopropanol (Sigma) was added and the tube was incubated at 4°C overnight. The next day, the tube was centrifuged at 3600 g for 60 min at 4°C. The supernatant was discarded and the pellet was resuspended and washed with ice-cold 70% ethanol (Sigma). After another centrifugation at 3600 g and 4°C for 10 min, the supernatant was completely but carefully removed using a fine Pasteur pipette. The tube was left upside down to air dry for 15 to 60 min until no liquid was visible. The dried pellet was resuspended with 25 μl of warm DEPC-H_2_O then placed on ice. RNA concentration was measured using a spectrophotometer. 12 μg of RNA from the R29 non-rosetting parasites at each of the six time points was combined together to form the reference pool. The pool and 12 μg of each individual time point sample from both rosetting and non-rosetting parasites were then used for first-strand cDNA synthesis using an amino-allyl dye coupling protocol [34].

### Printing the VSA-supplemented microarray chip

The microarray for the 3D7 *P. falciparum *genome designed by Hu *et al *[26], consists of 10,166 70-mer long oligonucleotide elements (oligos) for 5,363 genes, with one unique oligo every 2 kb per gene. To these were added oligos specific for the VSA from HB3, Dd2 and IT strains. Oligos were spotted onto polylysine-covered slides and post-processed as described in [35,36].

### cDNA labelling and microarray hybridization

Microarray hybridizations were performed as previously described [35]. Briefly, each aminoallyl-cDNA sample was coupled to Cy5 (red dye) while Cy3 (green dye) was added to the pool. Cy5-labelled time point samples were mixed with the same amount of Cy3-labelled pool sample. The solution was loaded on a microarray slide and hybridized for 14-16 h using a Maui hybridization system (Bio Micro Systems) at 65°C. Microarrays were scanned with a GenePix 4000B scanner (Axon Instruments).

### Pre-analysis and quality control

All arrays were visually inspected using GenePix and any poor quality spots (signal below background or dust on the chip) were flagged out. After gridding, the data were loaded onto the Acuity 4.0 software. Within this database, each spot was expressed as:

'F' is the foreground signal intensity, 'B' is the background signal intensity, '635' is the wavelength of the red channel, '532' is the wavelength of the green channel. Thus, the data for each spot is the ratio between red and green signal. Each array was then normalized with Lowess (locally weighted least squares regression). A dataset with all time points was created using the following parameters:

Cutoff: Discard flagged spots AND (% > B532+2SD > = 95 OR% > B635+2SD > = 95). In other words, only unflagged spots and spots with median intensities (green or red) greater than the local background plus two times the standard deviation of the background were used.

### Microarray data analysis

Expression ratios correspond to the "red signal" (from a rosetting R+ or non-rosetting R- time point sample) divided by the "green signal" (from the pool). These ratios were used to visualize the timing of expression of a particular gene. To measure the change of expression in R+ compared to R-, the [rosetting/pool] ratios were divided by the [non-rosetting/pool] ratios to obtain [rosetting/non-rosetting] ratios. Values from oligos specific to the same genes were averaged using OligoAverage.pl [26]. Data analysis was carried out using Microsoft Excel, Cluster [37] and Jalview [38] for data visualization. All microarray data have been deposited in the GEO repository [39]. Genes showing a three-fold change between the rosetting R+ and non-rosetting R- parasites in at least one of the five paired time points (TP1-5) were subjected to K-means clustering of groups. Data from TP6 were not examined further due to synchronization differences between R+ and R- at this time point. Functional Enrichment Analysis was carried out to calculate the over-representation of genes belonging to functional groups for each cluster as compared to their respective frequency in the whole genome.

## Results

### All variant surface antigen sequences from HB3/Dd2/IT were extracted to design specific microarray probes (70-mer oligos)

The software HMMer was used to extract all VSA sequences from HB3, IT and Dd2 genomes (Table 1). *Rif*, *stevor *and *pfmc-2tm *sequences are available as supplementary data (Additional files 1, 2, 3, 4). The *var *gene sequences from these three strains have been published by other groups [40,41]. A total of 990 70-mer oligos corresponding to the *var*, *rif *and *stevor *gene families of HB3, Dd2 and IT (Additional file 5) were added to the 10,166 existing oligos on the *P. falciparum *microarray chip [26]. Strain-specific oligos to *pfmc-2tm *were not included because the hypervariable loop of this gene family is only about 50 nucleotides long [42]. *Surfin *genes were first described as a VSA family [43], however ClustalW alignments with 3D7, HB3 and Dd2 *surfins *indicate that each orthologue is well-conserved between strains. Thus *surfin *is not a VSA family and there is no need to design new oligos for microarray purposes.

**Table 1:** Total number of gene sequences for each VSA family for each parasite strain

		3D7	HB3	Dd2	IT
***var***	genes	62	38	39	49
	Pseudogenes^a^	1	6	4	3
	Truncated genes^b^	0	6	7	4
	**Total**	**63**	**50**	**50**	**56**

***rif***	genes	153	100	99	102
	Pseudogenes	28	21	21	15
	Truncated genes	3	4	13	8
	**Total**	**184**	**125**	**132**	**125**

***stevor***	*stevor *genes	30	17	24	25
	Pseudogenes	7	9	7	8
	Truncated genes	0	0	0	0
	**Total**	**37**	**26**	**31**	**33**

***pfmc-2tm***	genes	10	6	2	5
	Pseudogenes	3	1	0	2
	Truncated genes	0	1	1	1
	**Total**	**13**	**8**	**3**	**8**

^a^Pseudogene: one or more stop codons or frameshifts within the sequence.

^b^Truncated gene: part of the gene sequence is missing, most likely due to incomplete sequencing.

### Some VSA sequences are conserved amongst strains

The similarities between the VSA from different parasite strains were examined. The *var *genes are not described further here because a detailed examination of the HB3/Dd2/IT *var *gene repertoires was reported recently [40]. Bioinformatic analysis of *rif *and *stevor *genes revealed that several of these VSA family members are conserved across the four analyzed strains. Those that were found in at least three out of the four strains with >90% amino acid identity between at least one pair are shown in Table 2 (13 strain-transcendent Rifins) and Table 3 (5 strain-transcendent Stevors). For comparison, a typical pairwise alignment score for two random Rifins is 30 to 50% amino acid identity. The average pairwise alignment score of all Rifins is alike for each strain (~ 44%) as well as all strains together (43.2%). This is in agreement with the hypothesis that each member of a VSA family can recombine with each other, possibly through heterologous meiotic crossing-over [44]. Pairwise alignment scores are in general higher for Stevors (~60%).

**Table 2:** Summary of strain-transcendent Rifins found in at least three out of 3D7, HB3, Dd2 and IT

3D7		HB3	Dd2	IT	**Pairwise alignment score**^**1**^					
**Gene ID**	**Found in F.I.**^**2**^	**Gene ID**	**Gene ID**	**Gene ID**	**3D7-** **HB3**	**3D7-** **Dd2**	**HB3-** **Dd2**	**IT-** **3D7**	**IT-** **HB3**	**IT-** **Dd2**

RifA2_PFD0070c		HB3rifA2_025	Dd2rifA2_p126^3^	IT4rifA2_125	92	83	85			

/		HB3rifB1_050	Dd2rifB1_027	IT4rifB_114			100		100	100

RifB_MAL13P1.495		HB3rifB_t022^4^	Dd2rifB_t038	IT4rifB_111	94	94	99	100	99	100

RifB_PFE1630w	yes	HB3rifB_079	Dd2rifB_081	/	99	85	87			

RifA_PFL2585c	yes	HB3rifA_098		IT4rifA_031	99			96	99	

RifA_PFD0645w		/	Dd2rifA_086	IT4rifA_021		98		94		95

RifA_p_PFD1020c	yes	HB3rifA_p112	/	IT4rifA_050	97			90	98	

/	yes	HB3rifA_051	Dd2rifA_p100	IT4rifA_052			99		100	98

RifB_p_PFA0710c	yes	HB3rifB_p116	Dd2rifB_p111	IT4rifB_104	97	98	99	98	99	98

RifA_PFE1635w	yes	HB3rifA_p115	Dd2rifA_p107	IT4rifA_p084	98	94	95	86	86	92

RifA_p_t_PFD0134c		HB3rifA_p101	/	IT4rifA_t072	100			95	90	

RifA_p_PF11_0022		/	Dd2rifA_p112	IT4rifA_p087		98		86		87

RifA_p_MAL7P1.52		/	Dd2rifA_p098	IT4rifA_p088		91		97		96

^1^Rifins with >90% amino acid identity between at least one pair of strains plus >80% amino acid identity between at least 2 other pairs of strains are shown.

^2^F.I: Brazilian field isolates [45]

^3^p=pseudogene: one or more stop codons or frameshifts within the sequence.

^4^t=truncated gene:part of the gene sequence is missing, possibly due to incomplete sequencing.

**Table 3:** Summary of strain-transcendent Stevors found in at least three out of 3D7, HB3, Dd2 and IT

3D7		HB3	Dd2	IT	**Pairwise alignment score**^**1**^					
**Gene ID**	**Found in F.I.**^**2**^	**Gene ID**	**Gene ID**	**Gene ID**	**3D7-** **HB3**	**3D7-** **Dd2**	**HB3 -** **Dd2**	**IT-** **3D7**	**IT-** **HB3**	**IT-** **Dd2**

SteA_MAL13P1.7		HB3steA_14	/	IT4steA_11	99			87	87	

SteB_PFD0125c		HB3steB_p18^3^	Dd2steB_18	IT4steB_01	99	93	91	99	93	92

SteB_t_PFB0955w^4^	yes	HB3steB_p26	/	IT4steB_14	98			99	99	

SteB_p_PF10_0009		HB3steB_p20	Dd2steB_p29	IT4steB_p31	98	98	98	88	88	88

SteA_p_PFA0705c	yes	HB3steA_p23	Dd2steA_p31	/	99	96	98			

^1^Stevors with >90% amino acid identity between at least one pair of strains plus >80% amino acid identity between at least 2 other pairs of strains are shown.

^2^F.I: Brazilian field isolates [45]

^3^p=pseudogene: one or more stop codons or frameshifts within the sequence.

^4^t=truncated gene:part of the gene sequence is missing, possibly due to incomplete sequencing.

**Figure 3:**
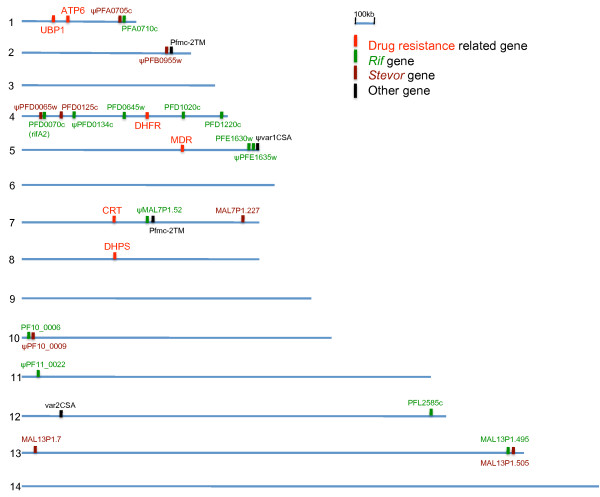
**Genomic location of strain-transcendent VSA in relation to drug-resistance genes**. The 14 *P. falciparum *chromosomes are represented to scale. The location of six drug resistance-related genes, 22 *rif *and *stevor *genes that are conserved between 3D7 and at least one other strain, two conserved *pfmc-2tm *genes, *var1csa *and *var2csa *are indicated. "ψ" indicates a pseudogene.

Comparison with data from field isolates in Brazil [45] showed that many of the conserved *rif *and *stevor *sequences are also found in field isolates (Tables 2 and 3). In several cases, conserved *rif *and *stevor *sequences are located in pairs next to each other in the subtelomeric regions (eg. chromosomes 1, 4, 10 and 13, Figure 3). When sufficient genomic data were available to allow examination of gene location, this synteny was conserved among strains. The internal non-coding DNA sequence in between such pairs of conserved genes was also conserved.

### The presence of conserved VSA cannot be explained by location next to known drug resistance genes

Previously, five *var *genes (*varS1*-*varS5*) were reported to be shared at relatively high frequency among field isolates from the West Pacific region [46]. Two of these genes, *varS2 *and *varS3 *were physically linked to the pyrimethamine-resistance *pfdhfr *locus (within 200 Kb), whereas another (*varS4) *was within 100 Kb of the chloroquine-resistance *pfcrt *locus. Thus, the fact that these *var *genes are conserved amongst isolates can be explained by a selective sweep due to heavy selection pressure for drug resistance in these parasites. A similar mechanism of VSA occurring adjacent to parasite genes that give a high survival advantage and undergo a selective sweep could in principle apply to the conserved VSA identified here. However, a chromosomal map reveals that few of the conserved VSA genes are located adjacent to known drug resistance genes (Figure 3). In addition, 3D7 and HB3 are sensitive to most of the commonly used anti-malarial drugs. Therefore, some other explanation is required to explain the presence of strain-transcendent *rif *and *stevor *genes.

### Time-course experiment with IT/R29 rosetting (R+) and non-rosetting (R-) parasites

**Figure 4:**
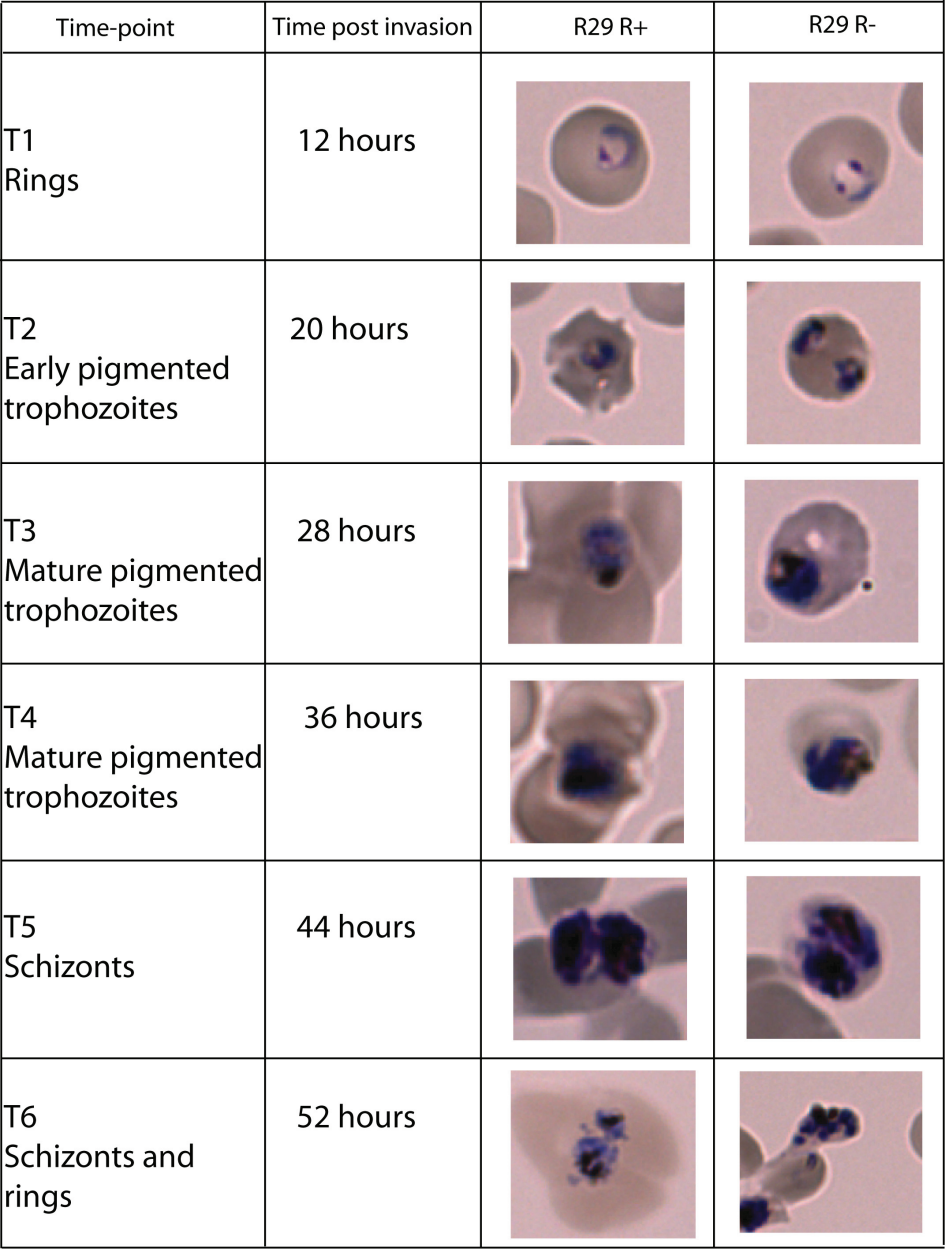
**Parasite maturity during the time-course experiment**. Sorbitol lysis was carried out at 12 hours after the first ring invasion was seen (time point one), and samples were then taken 8-hourly throughout the asexual blood stage cycle. The maximum parasite maturity in terms of hours post invasion at each time point is shown in the second column. Samples for RNA extraction were taken from the culture at each time point, mixed with TRIzol reagent and frozen, and a Giemsa-stained thin blood smear was performed to record the developmental stage of rosetting (R29R+) and non-rosetting (R29R-) parasites.

As proof of concept of the ability of the VSA-supplemented microarray chip to identify genes important in cytoadherence, we compared the whole transcriptome of IT/R29R+ parasites (rosette frequency 73.2%) with IT/R29R- parasites (rosette frequency 1.3%). RNA was collected from synchronized rosetting and non-rosetting parasites at six time points throughout the asexual blood stage cycle. The maturity of the two parasite populations was compared by Giemsa smear (Figure 4) and found to be similar throughout, apart from minor differences at time point six in which there were more rings and fewer schizonts in R+ parasites. In addition, statistical evaluation of gene transcript levels at each time point was carried out by Pearson correlation comparing data from all oligos in IT/R29R+ with all oligos at the same time point in IT/R29R-. A strong positive correlation was found (correlation coefficients above 0.88 for time points 1 to 5; 0.75 at time point 6), indicating that the two parasite populations were at similar levels of maturity. Therefore specific differences in gene expression detected in subsequent analyses are unlikely to be artefacts due to maturity differences between rosetting and non-rosetting parasites.

### *ITvar9 *is the only VSA highly up-regulated in IT/R29 rosetting parasites

**Figure 5:**
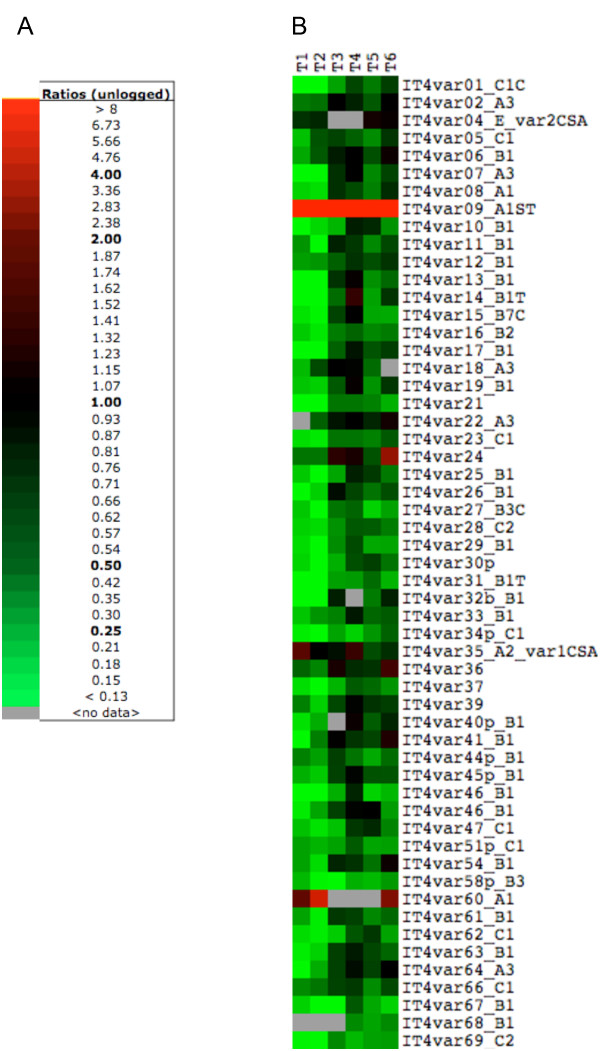
**IT/R29 *var *gene transcription in rosetting versus non-rosetting parasites analysed by VSA-supplemented microarray**. (A) Colour legend used for the microarray data. (B) Each row represents one *var *gene and values from multiple oligos specific to the same gene were averaged. Each *var *gene is represented by 2 to 5 oligos on the chip, with an oligo approximately every 2 kb for each gene. Each column is a time point with 8-hour intervals. *Var *gene names and groups (A, B or C) are as described previously [40,41]. Data represented are the expression ratio of data from IT/R29R+ over IT/R29R-, thus red squares indicate higher amount of cDNA in the IT/R29R+ compared to the IT/R29R- population, and green squares indicate less cDNA in IT/R29R+ than IT/R29R-. A grey square indicates the lack of data (expression intensity below background level). *Var *genes with expression intensity below background level at all time points are not shown. *ITvar9 *is up-regulated in IT/R29R+ by 19, 21, 115, 57, 109 and 439 fold from time point 1 to 6, respectively.

As the parasite ligand(s) mediating rosetting should be located on the surface of infected red cells, VSA are prime candidates for this function. For IT/R29R+, PfEMP1 encoded by *ITvar9 *(also called *R29var1*) is known to be the parasite rosetting ligand [22,23], although the existence of accessory molecules for rosetting has not been excluded. Examination of the *var *gene data from the microarray (Figure 5) showed that 55 out of the 56 annotated *var *genes in IT were transcribed, but only one, *ITvar9*, was highly up-regulated in IT/R29 rosetting parasites compared to non-rosetting parasites (19 to 439 fold up-regulated, Figure 5. By "up-regulated " we mean that the amount of mRNA for a particular gene is increased in IT/R29R+ compared to IT/R29R- parasites). This striking upregulation of *ITvar9 *was seen at all six time points. In the *var *gene family, the only other member to be up-regulated by more than two fold in rosetting parasites was *ITvar60 *(up to five fold up-regulated at time point 2), which is also a rosette-mediating variant in the IT strain (Ghumra and Rowe, in preparation). Virtually all other *var *genes were downregulated (i.e. expressed at a lower level in R29R+ than in R29R-, Figure 5). Therefore these data are consistent with previously published results indicating that *ITvar9 (R29var1) *encodes the ligand mediating rosetting in IT/R29 parasites [22,23], and authenticate the use of the VSA-supplemented microarray chip to detect important cytoadherence-associated gene candidates.

**Figure 6:**
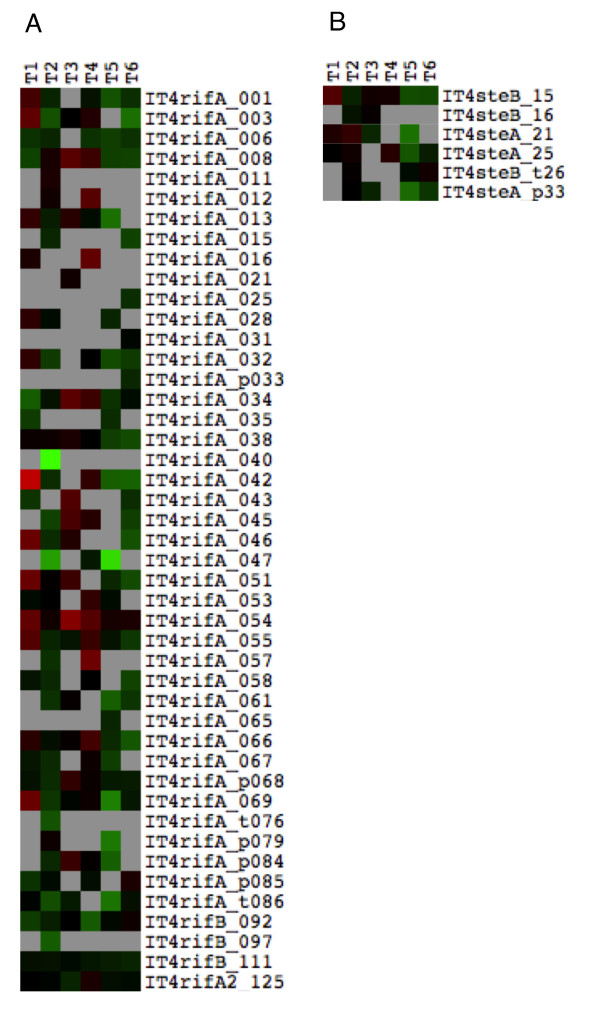
**IT/R29 *rif *and *stevor *gene transcription in rosetting versus non-rosetting parasites analysed by VSA-supplemented microarray**. (A) *Rif *genes and (B) *stevor *genes. The microarray data are as described in the legend for Figure 5. There was only one oligonucleotide probe per gene for *rif *and *stevor *genes due to their small size.

Whole transcriptome analysis with the VSA-supplemented chip also has the potential to identify accessory molecules for cytoadherence phenotypes. Analysis of the other VSA families in R29R+ and R29R- showed that only 45 *rif *and six *stevor *genes showed expression above background level in at least one time point, out of a total of 125 and 33 genes in the IT genome, respectively (Figure 6). This contrasts with the *var *gene family where almost all variants were transcribed (Figure 5). In the *rif *family, only *IT4rifA_042 *showed upregulation in rosetting parasites by more than four fold in a single time point (five fold at time point 1). Interestingly, *IT4rifA_054 *(alias *Rif13-1*), located upstream of *ITvar9 *in a "head to head" manner [47], is up-regulated by up to three fold in time point 3 (Figure 6A). This upregulation may result from the high transcription level of its neighbouring gene *ITvar9. *No *stevor *or *pfmc-2tm *variant was up-regulated by more than two fold in IT/R29R+ (Figure 6B).

Taking all VSA data together, *ITvar9 *is by far the most highly up-regulated VSA gene in R29 rosetting parasites, and these data do not support the involvement of other VSA families in rosette formation in this strain.

### Some non-VSA genes are up-regulated in IT/R29R+ parasites

Although only surface proteins could act as a direct rosetting ligand, other proteins could contribute to the phenotype, for example by influencing the trafficking of PfEMP1. Such non-VSA genes could also be up-regulated after rosette selection. As rosetting begins at the late ring/early trophozoite stage, genes expressed at time points 1 and 2 are likely to be the most relevant, although the possibility of relevant genes expressed later in the cycle cannot be excluded. Examination of the dataset showed that 377 genes (7.8%) were differentially expressed by at least three fold in rosetting compared to non-rosetting parasites (a full list of the differentially regulated genes is shown in Additional file 6). 47 of the 377 differentially regulated genes in rosetting parasites had a PEXEL motif, indicating export to the infected red cell cytoplasm [48]. Furthermore, a total of 17 genes belonging to the PHIST (*Plasmodium *helical interspersed subtelomeric) family were up-regulated. This gene family could be involved indirectly in the process of rosetting, as it is linked to export to the erythrocyte and host cell remodelling.

**Figure 7:**
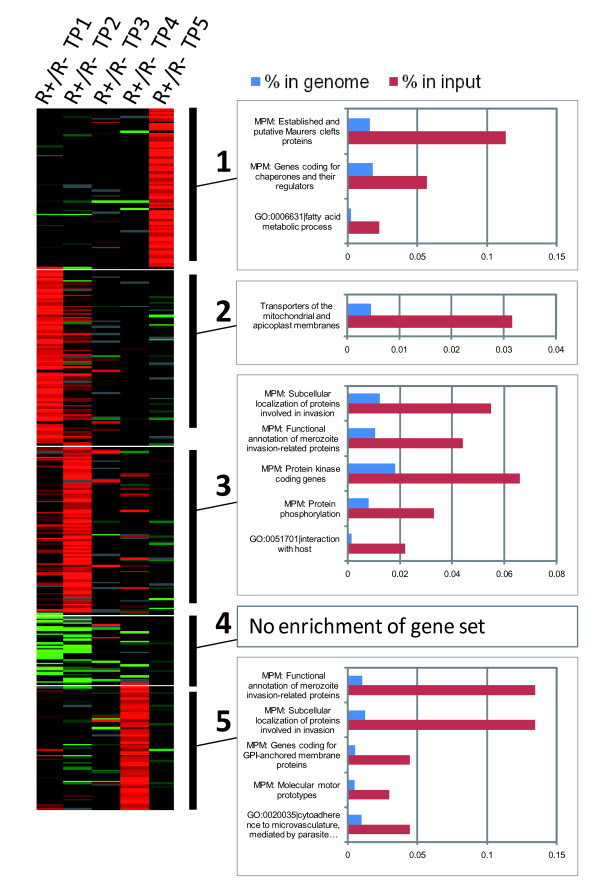
**Differentially expressed genes in rosetting and non-rosetting IT/R29 parasites**. The k-means clusters derived from the 377 genes that were differentially expressed by three-fold or more in rosetting compared to non-rosetting parasites are shown (colours as in Figure 5A). The corresponding graphs represent the enriched functional groups (p < 0.05) showing the over-representation of genes belonging to functional groups for each cluster as compared to their respective frequency in the genome as a whole.

K-means cluster analysis of the differentially regulated genes resulted in five distinct clusters in relation to timing of expression (Figure 7 and Additional file 6). Gene functional groups that were significantly enriched in rosetting parasites within each cluster were examined using three types of pathway, namely Gene Ontology (GO), Kyoto Encyclopedia of Genes and Genomes (KEGG) and Malaria Parasite Metabolic Pathways (MPMP). At time points 1 and 2 (clusters 2 and 3, Figure 7), enriched gene groups included mitochondrial transporters, invasion-related genes and protein kinases (the enriched genes within each cluster are shown in Additional file 6). A different set of invasion-related genes were up-regulated at time point 4 (cluster 5, Figure 7). Some cytoadherence-related genes were up-regulated at time point 4 (cluster 5, Figure 7), such as the cytoadherence linked asexual proteins 3.1, 3.2 and 2. At time point 5 (cluster 1, Figure 7), a significant enrichment of genes coding for established and putative Maurer's clefts proteins was observed. These include, MAHRP, Skeletal-binding protein, and several etramps. However, in all cases, the upregulation of the above genes enriched in rosetting parasites was modest in comparison to the upregulation of *ITvar9 *encoding the known rosetting ligand.

## Discussion

VSA sequences from HB3, Dd2 and IT genomes were extracted using HMMer software and Perl scripts. The multiple controls carried out using HMMer (repeating the approach with the output sequences and extraction of the original 3D7 VSA) give confidence that no VSA sequences were missed. The number of *var *genes sequences identified here matches or exceeds those identified previously using other data extraction methods [40,41]. Some *rif *and *stevor *sequences have been described previously for HB3 and IT [49], but not the entire repertoire. The possibility that some VSA genes are missing altogether from these sequenced genomes cannot be excluded, although the good sequence coverage (>9X) suggests that the gaps are likely to be small. Recently, Joannin *et al *released *rif *and *stevor *sequences from HB3 and Dd2 (not IT) on varDB [50]. They described more *rif *genes than this study, however, these extra sequences are truncated duplicates of other *rif *genes, typically found on short contigs. Considering the HB3 and Dd2 genomes are not fully assembled, these short duplicated sequences may well be assembly errors.

The VSA sequences were extracted in order to design unique oligos for each sequence to generate a VSA-supplemented microarray chip. However, further examination of the sequences revealed that several *rif *and *stevor *genes are well-conserved across strains (Tables 2 and 3). Almost half of these "conserved" VSA are predicted pseudogenes. Strain-transcendent *stevor *genes have been described previously [42,45,51], however, with the exception of *rifA2 *[49,50], this is the first comprehensive description of strain-transcendent *rif *genes. Two well-conserved strain-transcendent *var *genes have been described previously, *var2CSA *involved in placental malaria [52] and *var1CSA *of unknown function [53,54]. Interestingly, two of the conserved *rif *genes occur adjacent to *var1CSA *(within 6kb, Figure 3). Furthermore, the intervening non-coding sequence between these genes is also well-conserved, suggested that specific chromosomal regions in the subtelomeres may be conserved between strains. A previous example of conserved *var *genes has been described in *P. falciparum *isolates from the western pacific [46]. These conserved *var *genes are physically linked to drug resistance genes (within 100-200 Kb) and may, therefore, have hitch-hiked along with the drug resistance genes during a selective sweep under high drug pressure [46]. Examination of the location of the conserved *rif *and *stevor *genes in relation to known drug resistance loci (Figure 3) does not support a similar explanation in this case. The reason why these *rif *and *stevor *genes (some of them pseudogenes) appear well-conserved across strains is unknown. It is possible that these conserved VSA are linked to other genes that are highly benefical for parasite fitness and have undergone a selective sweep. Further work is needed to investigate this intriguing observation.

The original *P. falciparum *microarray chip [27] is only useful for the analysis of VSA from the 3D7 parasite strain on whose genome it is based. To allow whole transcriptome analysis (including VSA) of additional strains, we supplemented the 3D7-based chip with probes for the VSA of three strains (IT, HB3 and Dd2). As proof that this VSA-supplemented chip could be used to identify candidate genes of importance in cytoadherence, the transcriptomes of IT/R29 rosetting (R+) and IT/R29 non-rosetting (R-) parasites were analysed in a time-course experiment over the intraerythrocytic cycle. By far the most highly up-regulated gene in rosetting parasites was *ITvar9 *(alias *R29var1*), part of the group A family of *var *genes, which was up to 429-fold increased in R+ compared to R- parasites (Figure 5). This was expected from previous data showing that DBL1α of the PfEMP1 variant encoded by *ITvar9 *binds red cells to mediate rosetting [22], and antibodies to ITvar9 inhibit rosetting with high potency [23]. Transcription of the *ITvar9 *gene in rosetting parasites was up-regulated at all six time points, suggesting either that the mRNA for this gene is produced throughout the intraerythrocytic cycle, or that it is maintained without degradation right through to schizogony. The only other consistently up-regulated *var *gene in R29R+ parasites was *ITvar60 *(up to five fold up-regulated, Figure 5*)*, which encodes another rosette-mediating PfEMP1 variant in IT-derived parasites (Ghumra and Rowe, in preparation). The VSA-supplemented microarray chip therefore successfully identified the known rosetting ligand in IT/R29 as the leading candidate gene, and also identified another IT rosette-mediating variant.

Whether other VSA such as Rifins or Stevor are also involved in IT/R29 rosetting has not been investigated previously. The VSA-supplemented chip allowed us to determine whether any *rif *or *stevor *genes are highly up-regulated after selection for rosetting. Although a few *rif *genes were found expressed at a higher level in IT/R29R+ than in IT/R29R- parasites, the slight upregulation (two to five fold for *IT4rifA_042*) does not correlate with the large difference in rosetting frequency between the two populations and is minor compared to the upregulation of *ITvar9*. For *stevor*, no gene was even two-fold up-regulated in rosetting parasites. These data do not support the hypothesis that *rif *or *stevor *genes play a role in rosette formation in IT/R29.

One interesting observation regarding *rif *genes was that the gene "head to head" with *ITvar9 (IT4rifA_054*, alias *Rif13-1*), was up-regulated up to three fold. The association in expression between group A *var *genes and their upstream *rif *gene has already been shown in 3D7 parasites using the *PfSir*2 knockout line [55] as well as in 3D7 and IT/FCR3 parasites selected for group A *var *gene expression with children's serum from a malaria endemic area [49]. This co-regulation may be because the neighbouring *var *and *rif *genes are under the control of a common promoter. In fact, it has recently been shown that a titratable factor activates the transcription of all VSA families [56].

The analysis of the microarray data of non-VSA genes revealed 377 genes that were differentially expressed in rosetting and non-rosetting parasites by three fold or more (Figure 7). Many of these genes have PEXEL motifs [48] and could potentially be involved indirectly in cytoadherence, for example via PfEMP1 trafficking to the infected red cell surface. Replicates of the microarray experiment and further investigations would be needed before drawing any conclusion regarding the role of these genes. Parasites selected for other adhesion phenotypes including binding to brain endothelial cells and platelet-mediated clumping are currently being investigated using similar techniques, and will provide further information on non-VSA genes up-regulated after selection. Mok and colleagues previously performed a microarray analysis, comparing the transcriptome of rosetting versus CD36-selected 3D7 parasites [34]. Apart from the strain-specific VSA genes, they identified six non-VSA genes up-regulated by at least five fold in rosetting parasites. Only three of them (SERA-5, RESA-2 and PFI1445w) were up-regulated in R29 rosetting parasites (by two fold in a single time point), hence there is little overlap between their data set and the one reported here.

## Conclusions

New technologies allow the sequencing of parasite genomes at unprecedented scales and many additional *P. falciparum *laboratory strains and field isolates are expected to be sequenced in the near future. The method presented here, VSA sequence extraction with HMMer and oligo design with OligoRankPick, followed by microarray analysis of parasites selected for a cytoadherence phenotype, is straightforward and could be applied to any new *Plasmodium *genome. One advantage of this approach over other methods for studying VSA and cytoadherence such as real-time PCR, is that the entire transcriptome can be examined in each experiment. This allows for the possible identification of novel cytoadherence-related gene candidates that could not be predicted on the basis of previous knowledge. The VSA-supplemented microarray chip therefore has the potential to shed new light on the role of VSA and non-VSA genes in cytoadherence mechanisms.

## Competing interests

The authors declare that they have no competing interests.

## Authors' contributions

AG performed the rosetting selection and time course. AC performed the bioinformatics analysis, the microarray hybridizations and the data analysis. SM and APG assisted with the microarray experiment and microarray analysis. ZB provided advice and expertise on microarray analysis. AR conceived the study. AG, AC and AR drafted the manuscript. All authors read and approved the final manuscript.

## Supplementary Material

Additional file 1 ***Rif *gene sequences from HB3, Dd2 and IT**. Click here for file

Additional file 2 ***Stevor *gene sequences from HB3, Dd2 and IT**. Click here for file

Additional file 3 ***Pfmc-2tm *gene sequences from HB3, Dd2 and IT**. Click here for file

Additional file 4 ***Rif *and *stevor *gene names and supercontig location**. Click here for file

Additional file 5 **All VSA oligos added to the 3D7 microarray chip**. Click here for file

Additional file 6 **Non-VSA genes differentially expressed in rosetting versus non-rosetting parasites**. Click here for file
